# Supplementary material for: Genomic Diversity of Hospital-Acquired Infections Revealed through Prospective Whole-Genome Sequencing-Based Surveillance
Source: mSystems. 2022 Jun 13;7(3):e01384-21. doi: 10.1128/msystems.01384-21 (PMC9238379; doi:10.1128/msystems.01384-21)
Supplement: TABLE S1 [file msystems.01384-21-s0001.docx]

**Supplementary Table 1.** Species and source distribution of 3,004 bacterial isolates.

| **Species Group** | **# Patients** | **# Isolates** | **Blood** | **Respiratory** | **Urinary** | **Stool** | **Tissue/Wound** |
| --- | --- | --- | --- | --- | --- | --- | --- |
| ***Acinetobacter* spp.** | **71** | **82** | **6** | **50** | **4** | **-** | **22** |
| *A. baumannii* | 37 | 45 | 2 | 28 | 3 | - | 12 |
| ***Burkholderia* spp.** | **11** | **12** | **2** | **9** | **-** | **-** | **1** |
| *B. cenocepacia* | 4 | 5 | - | 4 | - | - | 1 |
| ***Citrobacter* spp.** | **118** | **126** | **10** | **29** | **37** | **-** | **50** |
| *C. freundii* | 39 | 42 | 4 | 9 | 12 | - | 17 |
| ***Clostridioides difficile*** | **458** | **501** | **-** | **-** | **-** | **501** | **-** |
| ST1 | 79 | 86 | - | - | - | 86 | - |
| ST2 | 62 | 63 | - | - | - | 63 | - |
| ST8 | 41 | 48 | - | - | - | 48 | - |
| ST42 | 39 | 40 | - | - | - | 40 | - |
| ***Enterococcus*spp. (VRE)** | **227** | **246** | **44** | **-** | **106** | **-** | **96** |
| *E. faecalis* (VREfs) | 8 | 8 | - | - | 2 | - | 6 |
| *E. faecium* (VREfm) | 221 | 240 | 44 | - | 106 | - | 90 |
| ST17 | 85 | 91 | 18 | - | 39 | - | 34 |
| ST736 | 31 | 32 | 9 | - | 18 | - | 5 |
| ST1471 | 27 | 30 | 5 | - | 8 | - | 17 |
| ST18 | 27 | 29 | 3 | - | 9 | - | 17 |
| ***Escherichia coli*(ESBL)** | **129** | **150** | **20** | **28** | **77** | **-** | **25** |
| ST131 | 89 | 103 | 16 | 19 | 51 | - | 17 |
| ST38 | 12 | 17 | 1 | 6 | 9 | - | 1 |
| ***Klebsiella*spp. (ESBL)** | **91** | **109** | **10** | **32** | **51** | **-** | **16** |
| *K. pneumoniae* (ESBL) | 82 | 97 | 10 | 29 | 43 | - | 15 |
| ST258 | 26 | 31 | 2 | 13 | 12 | - | 4 |
| ST405 | 14 | 16 | 2 | 5 | 7 | - | 2 |
| ST307 | 11 | 12 | - | 5 | 6 | - | 1 |
| ***Proteus* spp.** | **139** | **151** | **8** | **25** | **71** | **-** | **47** |
| *P. mirabilis* | 123 | 130 | 8 | 19 | 62 | - | 41 |
| ***Providencia* spp.** | **13** | **13** | **6** | **6** | **1** | **-** | **-** |
| *P. stuartii* | 6 | 6 | - | 2 | 1 | - | 3 |
| ***Pseudomonas aeruginosa*** | **653** | **863** | **52** | **436** | **213** | **-** | **162** |
| ST253 | 42 | 52 | 2 | 26 | 12 | - | 12 |
| ST179 | 30 | 40 | 4 | 26 | 3 | - | 7 |
| ST244 | 26 | 38 | 14 | - | 18 | - | 6 |
| ST27 | 25 | 34 | 3 | 19 | 3 | - | 9 |
| PA7/Group 3 | 11 | 15 | 0 | 11 | 3 | - | 1 |
| ***Pseudomonas* spp.** | **25** | **28** | **4** | **16** | **6** | **-** | **2** |
| ***Serratia*spp.** | **163** | **180** | **21** | **93** | **21** | **-** | **45** |
| *S. marcescens* | 160 | 177 | 21 | 92 | 21 | - | 43 |
| Clade A | 42 | 47 | 7 | 22 | 5 | - | 13 |
| Clade B | 24 | 27 | 3 | 15 | 4 | - | 5 |
| Clade C | 53 | 57 | 2 | 35 | 7 | - | 13 |
| Clade D | 19 | 21 | 5 | 10 | 2 | - | 4 |
| Clade E | 25 | 25 | 4 | 10 | 3 | - | 8 |
| ***Staphylococcus aureus*(MRSA)** | **358** | **420** | **70** | **188** | **13** | **-** | **149** |
| ST8 | 171 | 188 | 38 | 57 | 3 | - | 90 |
| ST5 | 120 | 149 | 15 | 85 | 9 | - | 40 |
| ST105 | 33 | 37 | 8 | 19 | 1 | - | 9 |
| ***Stenotrophomonas*spp.** | **106** | **123** | **7** | **92** | **20** | **-** | **4** |
| *S. maltophilia sensu strictu (Sm6)* | 43 | 57 | 1 | 45 | 2 | - | 9 |
| **Total** | **2562** | **3004** | **260** | **1004** | **620** | **501** | **619** |

ST = multi-locus sequence type; VRE = vancomycin-resistant enterococci; ESBL = extended-spectrum beta-lactamase-producing; MRSA = methicillin-resistant *S. aureus*.
