# Supplementary material for: Genomic Diversity of Hospital-Acquired Infections Revealed through Prospective Whole-Genome Sequencing-Based Surveillance
Source: mSystems. 2022 Jun 13;7(3):e01384-21. doi: 10.1128/msystems.01384-21 (PMC9238379; doi:10.1128/msystems.01384-21)
Supplement: FIG S1 [file msystems.01384-21-s0006.pdf]

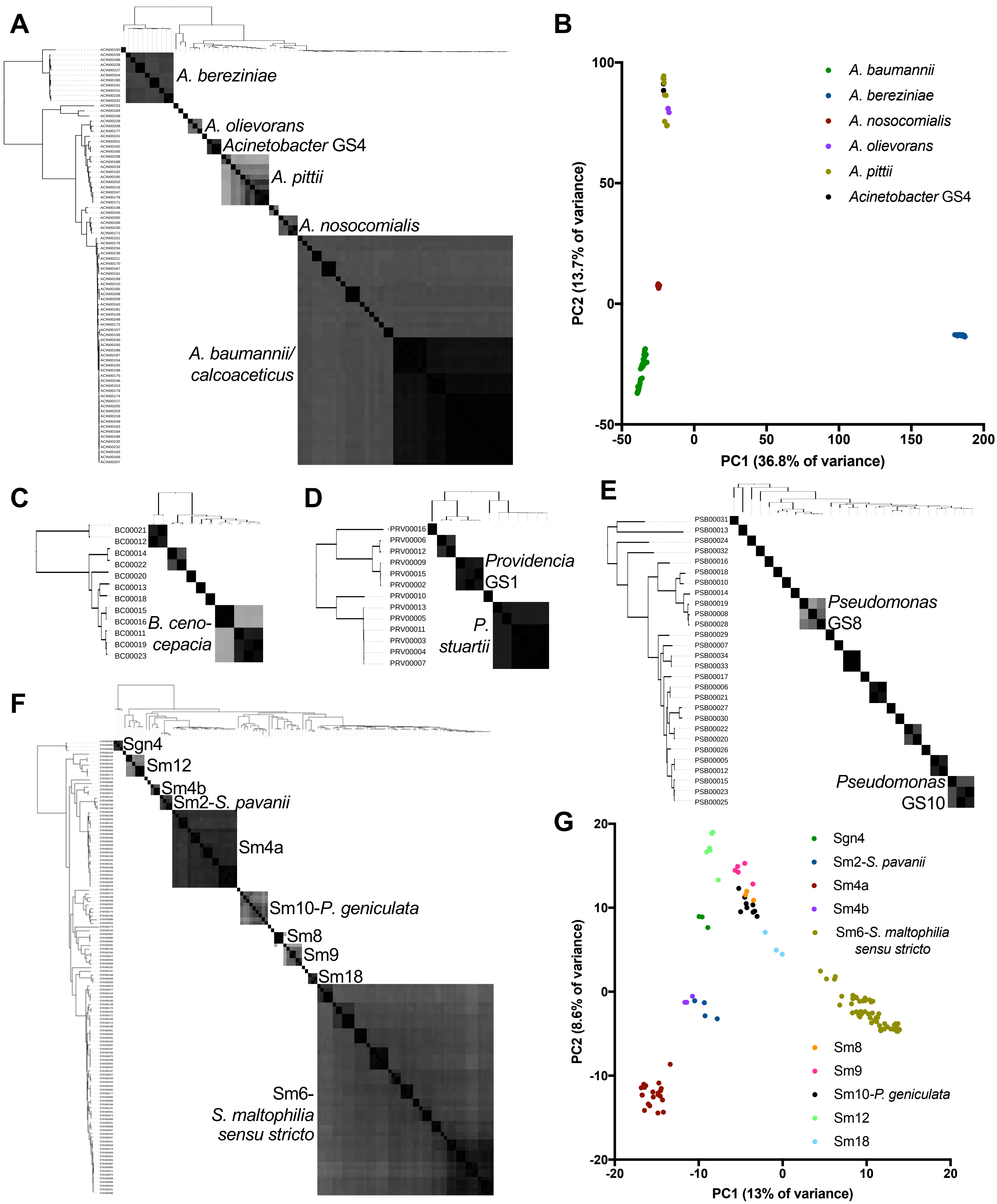

Fig. S1. Average nucleotide identity (ANI) and principal components analysis of accessory genes (PCA-A) among diverse species groups sampled by EDS-HAT. (A) Phylogenetic tree with pairwise ANI values and (B) PCA-A plot for *Acinetobacter* spp. (C) Phylogeny and ANI of *Burkholderia* spp., (D) *Providencia* spp., (E) *Pseudomonas* spp., and (F) *Stenotrophomonas* spp. (G) PCA-A plot for *Stenotrophomonas* spp. Grey shading indicates ANI values >95%, with darker shading showing higher identity. PCA-A plots include species with >2 isolates.
