# Supplementary material for: Genomic Diversity of Hospital-Acquired Infections Revealed through Prospective Whole-Genome Sequencing-Based Surveillance
Source: mSystems. 2022 Jun 13;7(3):e01384-21. doi: 10.1128/msystems.01384-21 (PMC9238379; doi:10.1128/msystems.01384-21)
Supplement: FIG S2 [file msystems.01384-21-s0007.pdf]

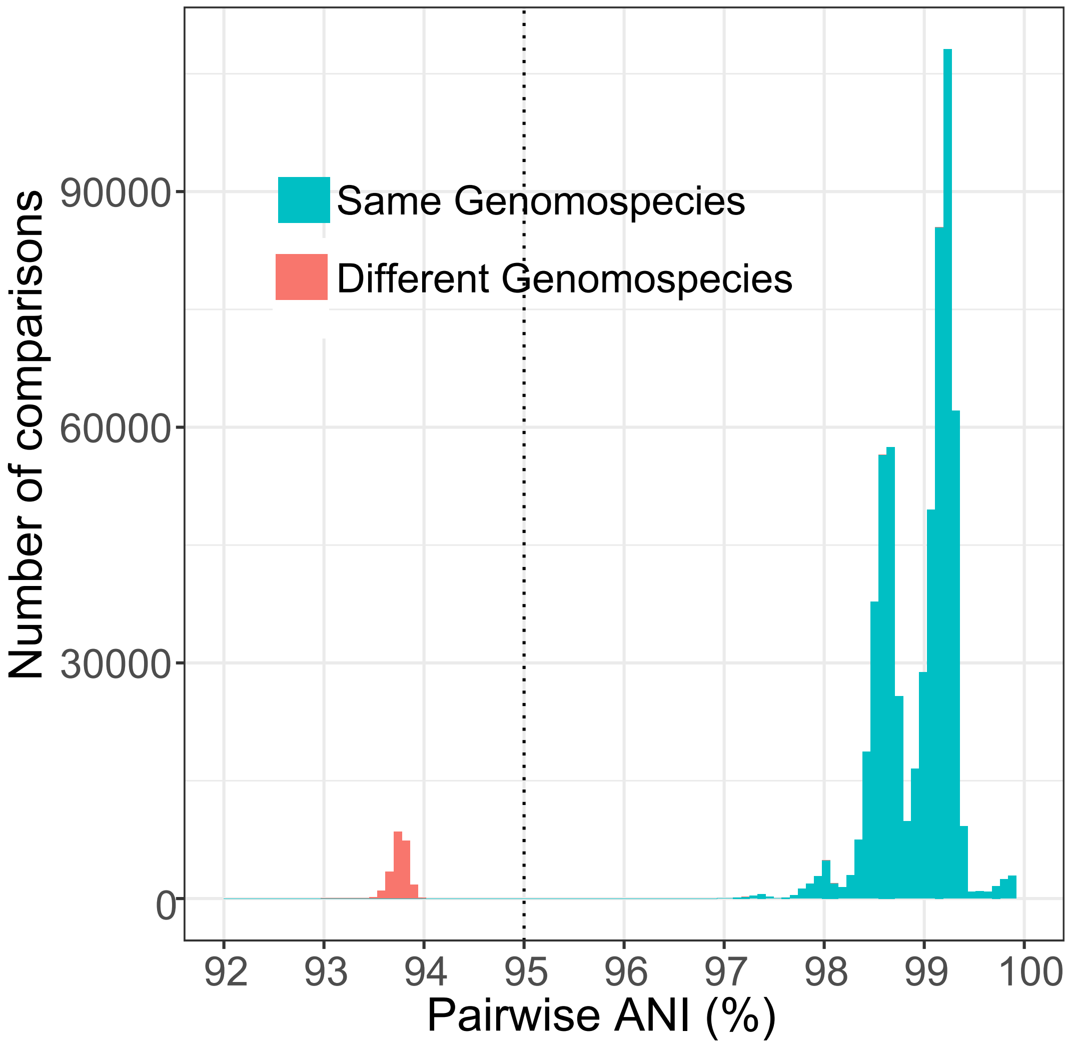

Fig. S2. Average nucleotide identity (ANI) comparisons of *P. aeruginosa* isolates. Histogram of pairwise ANI values for 863 *P. aeruginosa* isolate genomes sampled by EDS-HAT. Dashed vertical line indicates 95% ANI. Comparisons in red are between isolates in *P. aeruginosa* Groups 1 or 2 versus isolates in the PA7-like Group 3, which appear to belong to a distinct genomospecies.
