# Supplementary material for: Genomic Diversity of Hospital-Acquired Infections Revealed through Prospective Whole-Genome Sequencing-Based Surveillance
Source: mSystems. 2022 Jun 13;7(3):e01384-21. doi: 10.1128/msystems.01384-21 (PMC9238379; doi:10.1128/msystems.01384-21)
Supplement: FIG S3 [file msystems.01384-21-s0008.pdf]

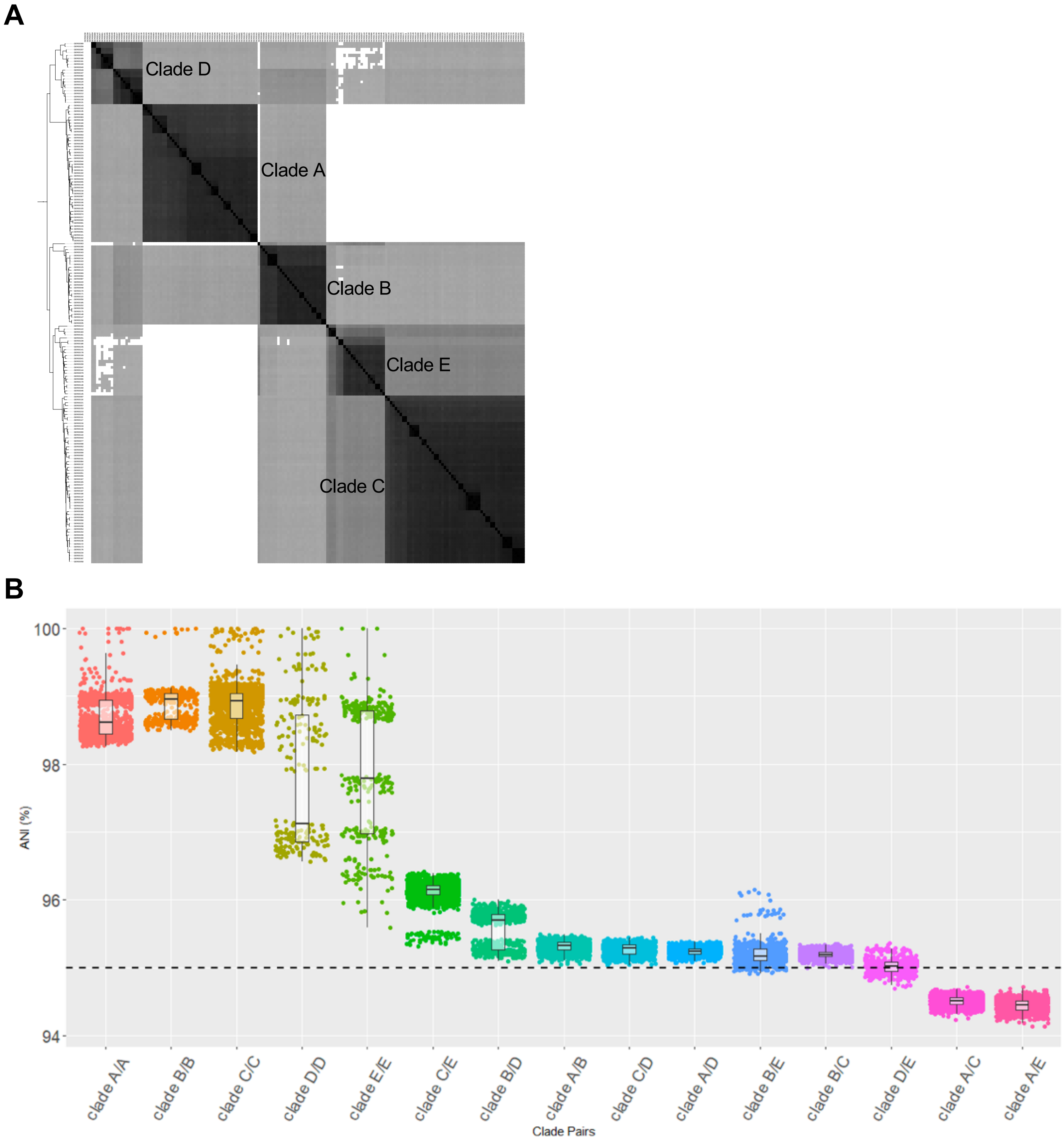

Fig. S3. Average nucleotide identity (ANI) comparisons of *S. marcescens* isolates. (A) Phylogeny and ANI of 177 *S. marcescens* isolates sampled by EDS-HAT. Grey shading indicates ANI values >95%, with darker shading showing higher identity. White indicates ANI values <95%. (B) Distribution of pairwise ANI values for *S. marcescens* isolates belonging to the same or different clades, broken down into pairwise clade comparisons. All comparisons between isolates in Clade A vs. Clade C and Clade A vs. Clade E fall below the standard species cutoff of 95%.
