# Supplementary material for: Genomic Diversity of Hospital-Acquired Infections Revealed through Prospective Whole-Genome Sequencing-Based Surveillance
Source: mSystems. 2022 Jun 13;7(3):e01384-21. doi: 10.1128/msystems.01384-21 (PMC9238379; doi:10.1128/msystems.01384-21)
Supplement: FIG S4 [file msystems.01384-21-s0009.pdf]

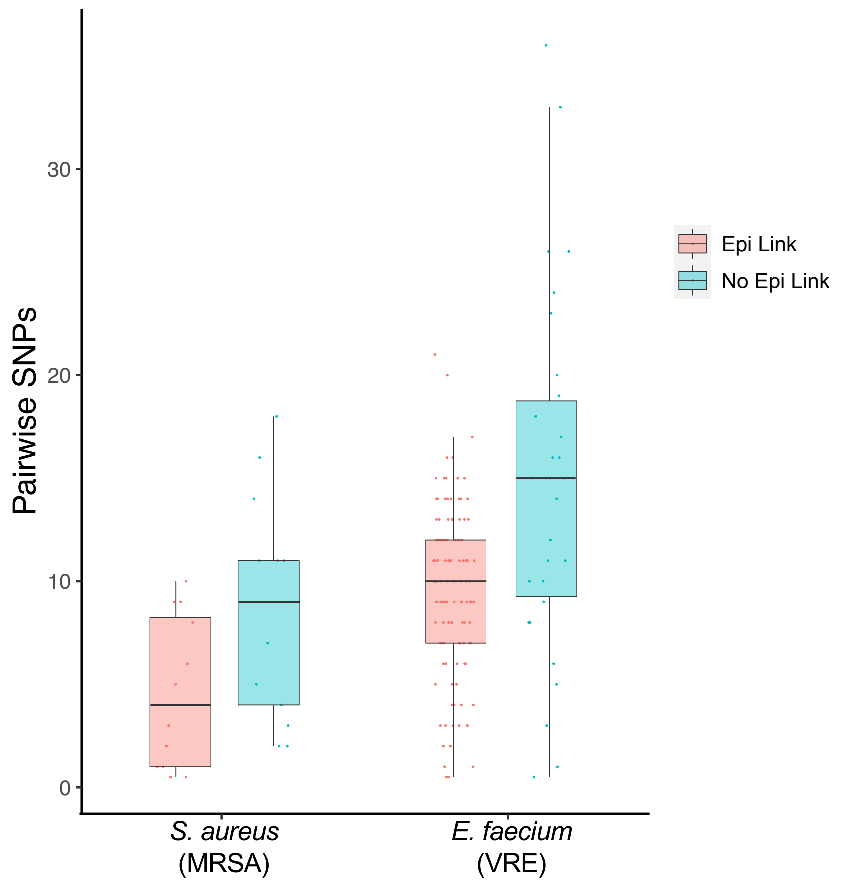

Fig. S4. Pairwise SNP comparisons of clustered isolates with and without epidemiologic links. Clusters of related isolates were identified using a SNP cut-off of 15 and single linkage clustering. Clusters were then grouped into those with identified epidemiologic links and those without. Pairwise SNPs are shown for all isolates in each cluster in each group. Boxes show the median, 25th and 75th percentiles.
