# Supplementary material for: Genomic Diversity of Hospital-Acquired Infections Revealed through Prospective Whole-Genome Sequencing-Based Surveillance
Source: mSystems. 2022 Jun 13;7(3):e01384-21. doi: 10.1128/msystems.01384-21 (PMC9238379; doi:10.1128/msystems.01384-21)
Supplement: FIG S5 [file msystems.01384-21-s0010.pdf]

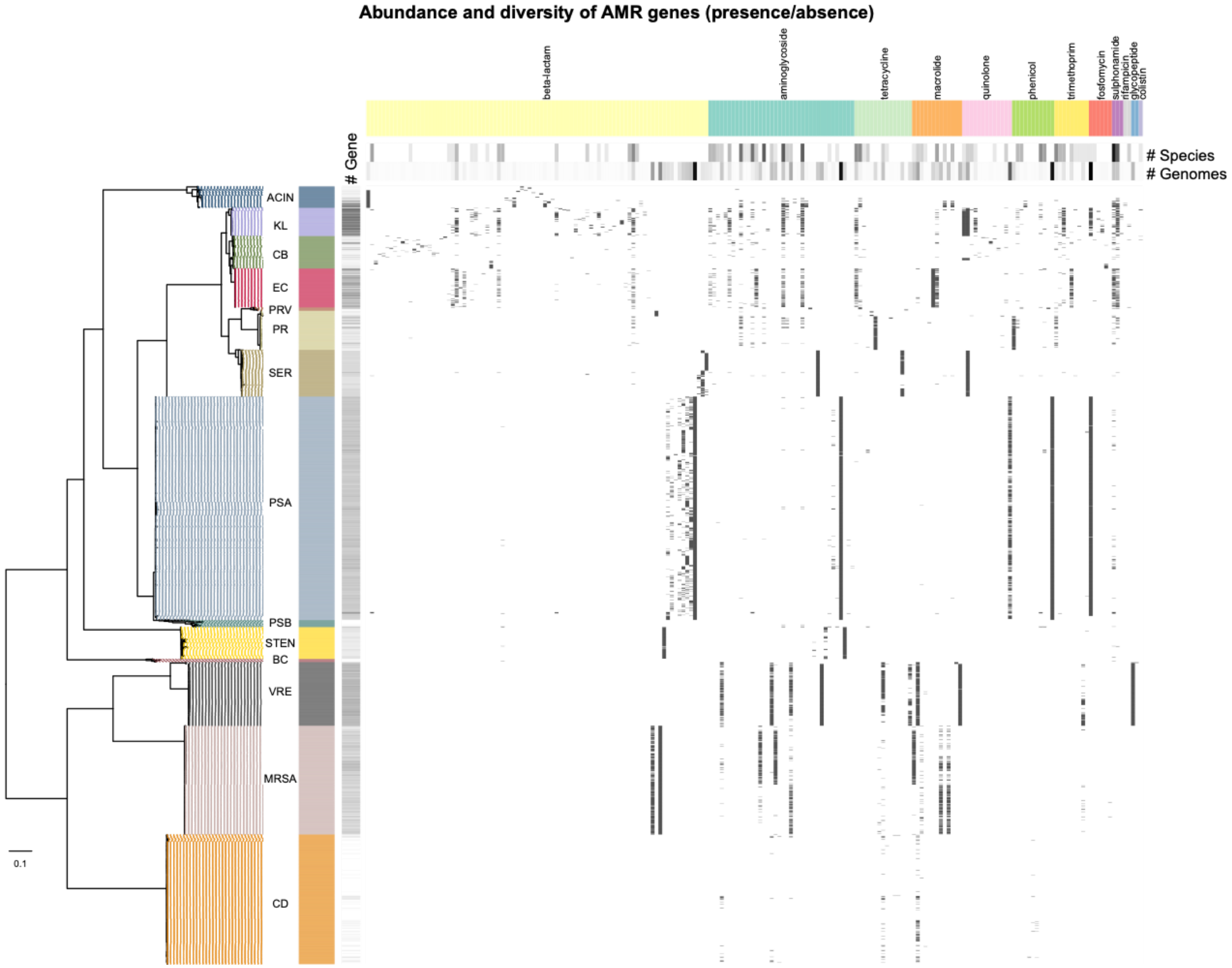


**Fig. S5. Distribution of antimicrobial resistance (AMR) genes among 3,004 clinical bacterial isolates from hospitalized patients.** Resistance genes were identified by BLASTn comparison to the ResFinder database. Isolates are ordered according to their phylogenetic placement using the amino acid sequences of 120 ubiquitous protein-coding genes from the Genome Taxonomy Database Tool Kit. "# Gene" shows the number of AMR genes per genome, with darker shading indicating more AMR genes. The matrix shows the presence or absence of 202 AMR genes, grouped by antibiotic class. Heat maps at the top show the number of species groups and total number of genomes encoding each gene, with darker shading indicating higher numbers. Raw data used to make the matrix are available in Table S3.
